# Quadratic Relationship Between Alexithymia and Interoceptive Accuracy, and Results From a Pilot Mindfulness Intervention

**DOI:** 10.3389/fpsyt.2020.00132

**Published:** 2020-03-10

**Authors:** Rachel V. Aaron, Scott D. Blain, Matthew A. Snodgress, Sohee Park

**Affiliations:** ^1^Department of Psychology, Vanderbilt University, Nashville, TN, United States; ^2^Department of Psychology, University of Minnesota Twin Cities, Minneapolis, MN, United States

**Keywords:** alexithymia, interoception, mindfulness meditation, interoceptive accuracy, interoceptive sensibility, emotional awareness, emotional granularity, affect labeling

## Abstract

Alexithymia, or a reduced ability to label and describe one's emotions, is a transdiagnostic construct associated with poor psychosocial outcomes. Currently, the mechanisms underlying affective deficits associated with alexithymia are unclear, hindering targeted treatment delivery. Recent research suggests deficient interoceptive awareness, or reduced awareness of one's internal bodily state, may be key in the etiology of alexithymia. It has long been demonstrated that mindfulness meditation can alter perceptions of one's own emotions and bodily cues. Therefore, it is possible that mindfulness meditation may reduce affective deficits associated with alexithymia by improving interoceptive awareness. In this study, we aimed to (1) elucidate the role of interoceptive accuracy and sensibility, two dimensions of interoceptive awareness, in alexithymia, and (2) test the efficacy of a brief mindfulness meditation for improving interoceptive accuracy, interoceptive sensibility, and emotional awareness. Seventy six young adults completed a baseline heartbeat detection task, to assess interoceptive accuracy and sensibility, and the Toronto Alexithymia Scale—20 item. They were randomly assigned to a brief mindfulness-based body scan meditation intervention or control condition. Afterwards, participants completed tasks assessing emotional awareness (i.e., affect labeling, emotional granularity) and follow-up heartbeat detection task. Relationships between alexithymia and interoceptive accuracy and sensibility were best described as quadratic (*p* = 0.002) and linear (*p* = 0.040), respectively. Participants in both conditions showed robust improvements in interoceptive accuracy from baseline to follow-up (*p* < 0.001; $\eta_{p}^{2}$ = 0.15); however, there were no group (meditation or control) differences in degree of improvement. Similarly, there were no group differences in affect labeling or emotional granularity. These preliminary results suggest that heightened alexithymia may be associated with either relatively high or low interoceptive accuracy. The meditation condition did not result in improved interoceptive accuracy or sensibility above and beyond that of a control group. Improvements in interoceptive accuracy, interoceptive sensibility, and emotional awareness may require longer or more interactive intervention approaches. More research is needed to parse the potentially complex relationship between alexithymia and interoceptive awareness, and to develop targeted treatment approaches to ameliorating associated affective deficits.

## Introduction

Alexithymia is a dimensional and transdiagnostic construct, defined by an inability to label and describe one's own emotional experiences, and a preference for externally-oriented thinking (1). Elevated alexithymia is problematic as it is associated with deficits in emotion regulation (2) and relatedly, elevated mental health risk (3). Alexithymia was once considered a stable personality trait; however, recent research suggests both targeted and general psychosocial interventions result in reductions of alexithymia (4–6). It is now exceedingly important to clarify the nature of alexithymic deficits and how to effectively target them in intervention. The current study takes steps toward this goal by investigating the relationship between alexithymia and interoceptive accuracy and sensibility, and piloting a mindfulness intervention to improve interoceptive accuracy, interoceptive sensibility and emotional awareness.

Interoception refers to one's perception of internal physiological sensations within their body (7). The capacity to accurately detect interoceptive cues is thought to be critical in facilitating the experience of subjective emotional states (8–10). Some propose that alexithymia is fundamentally a deficit in interoceptive accuracy (10–12). Thus, clarifying the relationship between alexithymia and aspects of interoception is essential. The umbrella of interoceptive awareness includes multiple related, but distinct processes including “interoceptive accuracy” and “interoceptive sensibility” (13). Interoceptive accuracy refers to one's *objective* accuracy in detecting interoceptive signals. In the laboratory, this is typically measured using heartbeat detection paradigms; for example, in Schandry's (14) mental tracking task, participants are instructed to estimate how many times their heart beats over various time intervals. Meanwhile, their actual heartrate is measured using a tracking device, allowing direct comparison of estimated and objective heartbeat data. The ratio of these values forms an index of interoceptive accuracy. Interoceptive sensibility refers to one's *perceived* dispositional tendency to focus on interoceptive signals, and is typically measured using self-report questionnaires that assess an individual's belief in their interoceptive ability (13). In the laboratory, this is often assessed by measuring one's confidence in performance on heartbeat detection paradigms.

Despite theoretical conjecture that alexithymia should be negatively related to interoceptive accuracy and sensibility, empirical findings testing this linear relationship are mixed. While some studies have shown significant negative relationships between alexithymia and interoceptive sensibility, assessed with self-report questionnaires (15, 16), others have reported significant positive relationships between alexithymia and interoceptive sensibility (17, 18).

Similarly, on heartbeat detection tasks, investigators have reported statistically significant negative [e.g., (12)], positive [e.g., (19)], and statistically non-significant relationships [e.g., (16)] between interoceptive accuracy and alexithymia. Murphey et al. (20) suggests inconsistent findings may result from failure to account for key covariates. They show alexithymia and interoceptive accuracy are significantly negatively correlated, but only after accounting for relevant covariates such as BMI [associated with lower interoceptive discrimination; (21)], depression and anxiety, and gender.

Another source of variance might be non-linear relationships between alexithymia and interoceptive accuracy and sensibility; consideration of specific clinical examples marked by elevated alexithymia raises this possibility. Some clinical examples are conceptually consistent with the theory that reduced interoceptive awareness relates to reduced emotional awareness. For example, eating disorders are characterized by elevated alexithymia (22) and abnormal internal bodily representations; it is perhaps unsurprising that individuals with eating disorders (compared to non-clinical controls) are characterized by interoceptive deficits (23). Other clinical populations do not present as consistent with this intuitive account. For example, compared to healthy controls, individuals with anxiety disorders have higher levels of alexithymia (3) but also increased interoceptive accuracy (24). Domschke et al. (24) argue that rather than provide useful knowledge about one's internal affective state, heightened interoceptive accuracy may create more frequent opportunities for catastrophic interpretation, perpetuating a cycle of anxiety. Longarz et al. (18) showed significant positive associations between alexithymia, interoceptive sensibility and an index of hypochondriasis. They conclude that heighted interoceptive sensibility can sometimes occur at the expense of emotionally relevant cues. Taken together, the literature suggests that reduced or elevated interoceptive accuracy or sensibility may be problematic in individuals characterized by difficulties with emotional awareness. This suggests the possibility of quadratic relationships between alexithymia and interoceptive accuracy and sensibility. Ultimately, understanding mechanisms underlying accurate, and healthy (e.g., non-catastrophic) interoceptive awareness may be key to ameliorating deficits associated with alexithymia.

Research highlights specific deficits in emotional awareness that are associated with alexithymia, and potentially modifiable in response to focused intervention. Alexithymia is associated with distinct patterns of labeling one's own emotions (i.e., affect labeling): in response to evocative images and videos, those with heightened alexithymia generate fewer or no emotion words to describe their feelings (25, 26). Alexithymia is also associated with reduced negative emotional granularity (26, 27), or the ability to make fine-grained distinctions between various negative emotional states. For example, an individual with low emotional granularity might describe feeling “bad,” while someone with high emotional granularity might describe the same feeling as “ashamed, embarrassed, and irritated” (28). Affect labeling and granularity are related to successful emotion regulation (28); knowing what one feels is critical for taking active steps to regulate that emotion (29). This link might help explain robust associations between elevated alexithymia and poor mental health outcomes (3). Critically, affect labeling and emotional granularity are modifiable and thus potential targets of intervention: affect labeling interventions have been shown to improve physiological recovery following a stressful speech task (30). Undergoing an 8-week mindfulness based stress reduction course has been shown to result in increased emotional granularity (31). In dialectical behavioral therapy (DBT), patients learn to increase awareness and acceptance of varied emotional states, which promotes successful emotion regulation (32).

An essential next step in alexithymia research is developing and testing targeted interventions to ameliorate specific deficits. A primary goal of mindfulness-based interventions is to cultivate non-judgmental and present focused awareness of internal experiences including bodily sensations, thoughts, and emotions (33); as such, mindfulness-based interventions, particularly those that are bodily-focused, may be ideally suited to foster accurate detection of both interoceptive and emotional cues. This is again demonstrated in DBT interventions, which use mindfulness-based approaches to increase emotional awareness (32). Results from two recent mindfulness-based interventions highlight the utility of applying these techniques to improve alexithymia specifically. In a large, non-clinical sample, Bornemann and Singer (6) showed increased interoceptive accuracy in response to a 9-month mindfulness-based training. These improvements were directly related to reductions in alexithymia over time. Edwards et al. (34) developed a brief mindfulness-based intervention delivered to individuals with high alexithymia, which resulted in more precise and complex affect labeling. In the current study, we examined the benefits of a brief mindfulness-based body scan meditation on interoceptive accuracy, interoceptive sensibility, and emotional awareness in a healthy population.

The current study aimed to (1) explore the relationship between alexithymia and interoceptive accuracy and sensibility in a sample of healthy young adults while accounting for key covariates of BMI and gender, and (2) pilot a brief mindfulness-based body scan intervention and determine whether it results in improved emotional awareness and interoceptive accuracy and sensibility, compared to a control task. To test our first aim, participants completed a heartbeat tracking task and an alexithymia questionnaire. We hypothesized alexithymia would be related to interoceptive accuracy and sensibility and we explored both linear and quadratic relationships. In the interest of transparency, it is important to acknowledge that although the heartbeat tracking task is widely utilized, recent empirical evidence highlights significant limitations of this method (35); we discuss the implications of these limitations in the discussion section. To address our second aim, we randomized participants to receive either a mindfulness-based body scan meditation intervention or to complete a control task. Similar meditations have been successful in increasing accurate perception of bodily sensations in prior research (36). We hypothesized that those in the mindfulness (vs. control) group would demonstrate greater improvements in interoceptive accuracy and sensibility from baseline to follow-up, and improved performance on affect labeling and emotional granularity tasks.

## Materials and Methods

### Participants

Participants were recruited from the university's undergraduate research pool. They responded to a general advertisement describing a laboratory visit entailing questionnaires and laboratory tasks. Seventy six participants were recruited and received course credit for their participation. To account for the potentially confounding effect of mood disorders on study variables, we excluded participants with a past or current self-reported diagnosis of depression, anxiety, or substance use disorder. This study was approved by the Vanderbilt University Institutional Review Board, and participants provided written informed consent.

### Procedure

Prior to attending a scheduled laboratory visit, participants were randomized to either a mindfulness-based body scan meditation intervention condition or a control condition using a random number generator. When they arrived to the laboratory, they first completed questionnaires and a heartbeat detection task (14), used to measure interoceptive accuracy and interoceptive sensibility. Next, they received either a mindfulness-based body scan meditation intervention or control condition, depending on prior randomization.

Both conditions were delivered via a 10-min audio recording, and participants were instructed to simply follow along with instructions in the recording. The mindfulness intervention conditions consisted specifically of a body scan mediation; participants were instructed to sequentially attend to specific parts of their body with non-judgmental and present-focused attention; in the control condition, participants were instructed to listen passively to a reading from a natural history text book. Both recordings were originally developed for use in a smoking cessation intervention (37). Afterwards, participants completed tasks designed to assess multidimensional aspects of emotional awareness [for details on this “INduction-based multiDimensional Emotional Experiences Paradigm” see previous work; (26)]. Finally, they completed a follow-up heartbeat detection task.

### Materials and Tasks

#### Questionnaires

Participants completed the Toronto Alexithymia Scale 20-item [TAS-20; (38)], which yields a total alexithymia score, summed from scores on three subscales, difficulty identifying feelings (DIF), difficulty describing feelings (DDF), and externally-oriented thinking (EOT). Participants responded to questions on a 1–5 Likert scale; higher scores indicate greater alexithymia. Participants also completed a brief demographics questionnaire to assess age, gender, race, height, and weight (the latter two used to calculate BMI).

#### Heartbeat Detection Task

Interoceptive accuracy and interoceptive sensibility were assessed using the heartbeat tracking task (14). A Polar H7 Bluetooth Smart Heart Rate Sensor (Polar Electro Oy, Kempele, Finland) was utilized to measure heartrate during the heartbeat tracking trials. Heartrate data was transmitted using Bluetooth technology to a 4th generation iPad using the Polar Beat App (Apple Inc., Cupertino, CA, USA). To avoid references to the passing of time, participants were asked to remove watches for the duration of the heartbeat detection task, which were stored in a safe place outside of the data collection room; all clocks were also removed from the room. Participants were instructed to sit with their eyes closed, hands on the table, and palms face up, to avoid using visual cues or pulse detection as a proxy for heartbeat. Participants were asked to estimate the number of times their heart beat over various time intervals. First, they completed a 30 s practice trial and were given the opportunity to ask questions. Next, the completed 25 s, 35 s, and 45 s trials, with trial order randomized across participants. After each trial, they were asked “how many times did your heart beat?” After completing all four trials, they rated confidence in their estimation on a 1–10 scale (1 = “not at all confident,” 10 = “completely confident”).

Interoceptive accuracy was calculated as the mean score across the three trials using the following transformation: 1/3 ∑[(1-|recorded heartbeats—counted heartbeats|)/recorded heartbeats; (14)]. Confidence ratings were used as an index of interoceptive sensibility (13). Participants were not given any feedback on their performance after completing the task.

#### Emotional Awareness Tasks

After the mindfulness intervention or control task, participants completed the INduction-based multiDimensional Emotional Experiences Paradigm [IN-DEEP; (26)] to assess aspects of emotional awareness. Participants viewed 14 film clips identified as reliably eliciting 6 prototypical emotions by Gross and Levenson [(39); amusement, anger, contentment, disgust, fear, sadness, and surprise]. After each clip, they selected the emotion they experienced “the most” from a list including 16 emotion words and two additional options of “the emotion I experienced is not listed here” and “I did not experience any emotion.” Consistent with our prior research (26), the frequency with which participants selected the standardized emotion was used to compute an index of “consistent affect label.” The frequency with which they chose an affect label that did not correspond with the standardized emotion was used to form an index of “non-standard affect label,” and the frequency of selecting “I did not experience any emotion” formed an index of “no affect label.”

After providing an affect label, participants were presented with 29 emotional words sequentially, and asked to report the intensity with which they experienced each on a 1–9 Likert Scale. These ratings were used to form an index of emotional granularity. We calculated the degree to which each participant made granular distinctions between specific negative and specific positive emotions using a standard approach (28). First, interclass correlations with absolute agreement were calculated between the intensity ratings of each individual's endorsement of all negative or positive emotions across the entire experiment. For each participant, these ICCs were averaged together to form a single index representing average positive or negative granularity. At this stage, a high average ICC suggests a tendency to rate all positive or all negative emotion words similarly, reflecting a lower degree of granularity. However, to aid interpretation of these results, and in line with standard procedures, we then subtracted the average ICC from one, such that higher scores reflect a higher degree of granularity. Finally, scores were transformed to Fisher's *z*.

Of note, the full IN-DEEP paradigm assesses other domains of emotional experiences (e.g., subjective arousal, response time). We isolated affect labeling and emotional granularity to facilitate group comparison: for these variables, higher scores indicate report of more normative experiences or greater degree of emotional awareness. These two variables have been shown to be most tightly linked to the alexithymia construct (26, 27).

### Data Analysis

#### Power Analysis

A power analysis was conducted to determine the number of participants needed to sufficiently power our primary aim (determining the relation between interoceptive accuracy and alexithymia). One previous study reported a correlation of −0.37 between alexithymia and interoceptive accuracy in a sample from the general population (12). Thus, approximately 73 participants were needed to have 90% power to detect a similar, moderate, effect.

#### Missing Data

Heart rate data was missing from three participants at both time points (due to equipment failure or preference not to participate), and two participants at follow-up (due to time constraints). These data points were removed case-wise from our analyses.

#### Statistical Approach

To test our first aim, hierarchical regression models were conducted to examine relationships between (1) alexithymia and interoceptive accuracy, and (2) alexithymia and interoceptive sensibility. We tested relationships between total alexithymia and alexithymia subscales in relation to interoceptive accuracy and sensibility. In all models, BMI and gender were entered as the first step in each model. In the second step, baseline interoceptive accuracy (model 1) or interoceptive sensibility (model 2) were added to the model. In the third step, the quadratic term of baseline interoceptive accuracy (model 1) or interoceptive sensibility (model 2) was added to the model. Step two yielded estimates for a linear model, and step three yielded estimates for a quadratic model. We examined the significance and effect size (*f*^2^) of the overall model at each step. We also examined the significance of the individual items within. Finally, we examined the change in the effect of the overall model and the statistical significance of that change at each step, to determine whether a quadratic model was a better predictor of alexithymia, compared to linear effects. Of note, our decision to test a quadratic relationship between interoceptive accuracy and sensibility and alexithymia was a posteriori, driven by literature that emerged after the planning of this study. For example, a meta-analysis from our group that concluded that chronic pain—a condition characterized by hypervigilance toward bodily sensations—was consistently and robustly associated with elevated levels of alexithymia (40). Because this was not an *a priori* analysis, there is increased risk of Type I error.

Our second aim examined whether exposure to mindfulness-based body scan meditation intervention resulted in improved interoceptive accuracy and sensibility, and greater emotional awareness, compared to a control task. We conducted mixed repeated measure analysis of variance (RM-ANOVA) to examine the effect of time (baseline, follow-up) on interoceptive accuracy and sensibility, considering group (meditation, control) as a between-groups factor. Where significant interactions emerged, we conducted *post-hoc* paired sample *t*-tests to examine group differences. Finally, we performed *t*-tests comparing group differences in affect labeling and positive and negative emotional granularity, and report Cohen's *d* to describe effect size.

## Results

### Descriptive

#### Sample Characteristics

Of the included 76 participants, 66% identified as women and 34% as men (Mean age = 19.70, *SD* = 0.95). Fifty four percentage of participants identified as Caucasian, 16% African American or African, 15% Asian or Asian American, 3% Hispanic, and 10% “Multiracial” or “other.” The meditation and control groups did not differ on these demographic factors.

#### Alexithymia

Mean TAS-20 total score was 44.57 (*SD* = 9.36). There were no group differences (meditation vs. control) on TAS-20 total score (*t*_(75)_ = 0.04, *p* = 0.97). There were significant correlations between TAS-20 subscales; subscale score means, standard deviations and inter-correlations coefficients are reported in Table 1.

**Table 1:** Descriptive statistics and inter-correlations of alexithymia subscale scores.

**Alexithymia subscale**	**Mean (SD)**	**Inter-correlations**		
		**1**	**2**	**3**
Difficulty describing feelings	11.88 (3.63)		**0.715**	**0.247**
Difficulty identifying feelings	14.92 (4.33)	**<0.000**		**0.238**
Externally oriented thinking	17.76 (4.15)	**0.032**	**0.038**	

*Values above the diagonal indicate correlation Pearson Correlation coefficient (r). Values below the diagonal indicate p values. Statistically significant values (p < 0.05) bolded*.

### Relationship Between Alexithymia and Interoceptive Accuracy and Sensibility

#### Interoceptive Accuracy

**Figure 1:**
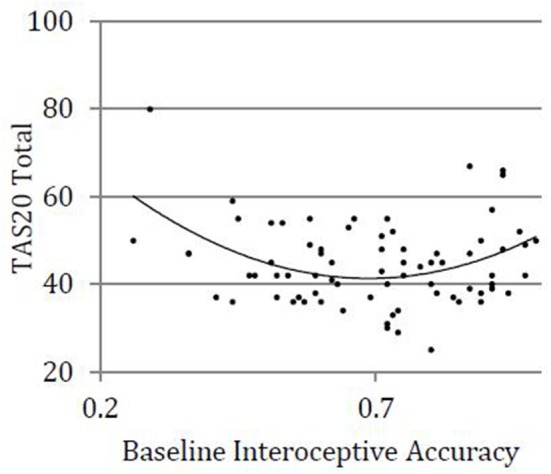
Quadratic relationship between alexithymia and interoceptive accuracy.

We used hierarchical regression models to examine linear and quadratic relationships between baseline interoceptive accuracy and total alexithymia, controlling for BMI and gender. See Table 2 for full results. The first step, including covariates (BMI, gender) was not statistically significantly related to total alexithymia (step 1; *R*^2^ = 0.02, *p* = 0.460). The second step, which added a linear interoceptive accuracy term was also not statistically significantly related to total alexithymia (step 2; *R*^2^ = 0.03, *p* = 0.612). The third step, which added a quadratic interoceptive accuracy term, was statistically significant (step 3; *R*^2^ = 0.17, *p* = 0.017) and moderate in effect (*f*^2^ = 0.20). The addition of a quadratic term to the overall model resulted in statistically significant improvement in *R*^2^, highlighting the statistical superiority of a quadratic vs. linear relationship between alexithymia and interoceptive accuracy (Δ*R*^2^ = 0.14, *p* = 0.002). Examination of individual items revealed that after accounting for covariates and the linear interoceptive accuracy term, there was a statistically significant quadratic relationship between interoceptive accuracy and alexithymia (β = 2.62, *p* = 0.002); see Figure 1. Of note, a single outlying TAS-20 score (TAS-20 = 80; greater than three standard deviations above the mean) was identified. We explored the quadratic relationship when this outlier was removed: After accounting for covariates and the linear interoceptive accuracy term, the relationship between interoceptive accuracy and alexithymia remained statistically significant (β = 1.89, *p* = 0.034).

**Table 2:** Hierarchical regression models of linear and quadratic relationships between alexithymia and baseline interoceptive accuracy.

		**β**	***t***	***p***	***R*^**2**^**	***p***	***f^**2**^***	**Change**	
								**Δ*R*^**2**^**	***p(*ΔF*)***
Step 1					0.02	0.460	0.02	0.02	0.460
	BMI	−0.16	−1.24	0.218					
	Gender	−0.07	−0.553	0.582					
Step 2					0.03	0.612	0.03	0.00	0.605
	BMI	−0.15	−1.14	0.259					
	Gender	−0.07	−0.54	0.594					
	IA_b_	−0.06	−0.52	0.605					
Step 3					**0.17**	**0.017**	**0.20**	**0.14**	**0.002**
	BMI	−0.09	−0.70	0.489					
	Gender	0.04	0.34	0.732					
	**IA**_**b**_	–**2.66**	–**3.35**	**0.001**					
	${\text{IA}}_{\text{b}}^{2}$	**2.62**	**3.30**	**0.002**					

*BMI, Body Mass Index; IA_b_, Baseline Interoceptive Accuracy. Statistically significant values (p < 0.05) bolded*.

We also conducted a series of models examining the relationship between alexithymia subscales and interoceptive accuracy, which yielded similar patterns of results. The relationship between DDF and DIF and interoceptive accuracy were best characterized as quadratic, effects which were moderate in magnitude. The relationship between EOT and interoceptive accuracy was also best characterized as quadratic, an effect which was small in magnitude. See Supplemental Material A for full results.

#### Interoceptive Sensibility

We conducted a similar series of models to examine relationships between baseline interoceptive sensibility and total alexithymia (Table 3). The first step, including covariates (BMI, gender) was not statistically significantly related to total alexithymia (step 1; *R*^2^ = 0.02, *p* = 0.478). The second and third step, which added first linear (step 2; *R*^2^ = 0.08, *p* = 0.124) and then quadratic (step 3; *R*^2^ = 0.09, *p* = 0.214) interoceptive sensibility terms, were not statistically significantly related to total alexithymia. However, examination of individual items revealed a statistically significant linear relationship between interoceptive sensibility and total alexithymia, after accounting for covariates (β = –0.26, *p* = 0.040). We explored this same relationship after removing the single outlying TAS-20 score (TAS-20 = 80), and the result was trending significance (β = –0.23, *p* = 0.074).

**Table 3:** Hierarchical regression models of linear and quadratic relationships between alexithymia and baseline interoceptive sensibility.

		**β**	***t***	***p***	***R*^**2**^**	***p***	***f^**2**^***	**Change**	
								**Δ*R*^**2**^**	***p(*ΔF*)***
Step 1					0.02	0.478	0.02	0.02	0.478
	BMI	−0.16	−1.21	0.230					
	Gender	−0.07	−0.53	0.597					
Step 2					0.08	0.124	0.09	**0.06**	**0.040**
	BMI	−0.18	−1.40	0.165					
	Gender	−0.02	−0.13	0.900					
	**IS**_**b**_	–**0.26**	–**2.10**	**0.040**					
Step 3					0.09	0.214	0.10	0.00	0.758
	BMI	−0.18	−1.43	0.159					
	Gender	−0.02	−0.12	0.902					
	IS_b_	−0.45	−0.71	0.480					
	${\text{IS}}_{\text{b}}^{2}$	0.19	0.310	0.758					

*BMI, Body Mass Index; IS_b_, Baseline Interoceptive Sensibility. Statistically significant values (p < 0.05) bolded*.

We also conducted a series of models examining the relationship between interoceptive sensibility and alexithymia subscales, which yielded similar overall patterns for DDF and DIF. Both relationships were best characterized as linear, although the relationship between subscales and the linear term were both trending significant (0.10 > *p* > 0.05). In contrast, there was no statistically significant relationship between EOT and interoceptive sensibility. See Supplemental Material B for full results.

### Change in Interoceptive Accuracy and Sensibility

Means and SD's of interoceptive accuracy and sensibility at baseline and follow-up by group are presented in Table 4.

**Table 4:** Interoceptive accuracy and sensibility means and standard deviations at baseline and follow-up.

	**Control group**		**Meditation group**	
	**Baseline**	**Follow-up**	**Baseline**	**Follow-up**
Interoceptive accuracy (%)	0.69 (0.17)	0.76 (0.14)	0.71 (0.18)	0.78 (0.16)
Interoceptive sensibility (0–10)	5.66 (1.77)	5.97 (1.66)	6.19 (2.07)	5.89 (1.98)

#### Interoceptive Accuracy

Mixed RM-ANOVAs examining change in interoceptive accuracy from baseline to follow-up revealed a main effect of time on interoceptive accuracy [*F*_(1, 70)_ = 12.22, *p* < 0.001], suggesting statistically significant and large in magnitude improvements in interoceptive accuracy from baseline to follow-up (η^2^ = 0.15). However, there was no interaction of time and group [*F*_(1, 70)_ = 0.02, *p* = 0.88, η^2^ = 0.00) on this relationship, suggesting that these improvements were statistically similar for both meditation and control groups.

#### Interoceptive Sensibility

There was no main effect of time on interoceptive sensibility [*F*_(1, 70)_ = 0.00, *p* = 0.96, η^2^ = 0.00], suggesting that there were no overall improvements in interoceptive sensibility, across the two groups. However, there was a significant time by group interaction [*F*_(1, 70)_ = 4.41, *p* = 0.039], an effect that was moderate in magnitude (η^2^ = 0.06). This suggests the presence of group differences (meditation vs. control) in changes in interoceptive sensibility from baseline to follow-up. We further explored this finding with *post-hoc* paired sample *t*-tests. Participants who received the mindfulness-based body scan intervention demonstrated a statistically non-significant reduction in interoceptive sensibility from baseline to follow-up [*t*_(34)_ = 1.25, *p* = 0.22, *d* = 0.15]. Those who received the control task recording demonstrated a statistically non-significant increase in interoceptive sensibility [*t*_(33)_ = −1.92, *p* = 0.063, *d* = 0.18].

### Group Difference in Emotional Awareness

There were no significant group differences on IN-DEEP performance between the control group and meditation groups (Table 5).

**Table 5:** Group differences (meditation intervention vs. control condition) in emotional awareness.

	**Control mean (SD)**	**Meditation mean (SD)**	***t***	***p***	***d***
Affect label					
Consistent affect label	9.18 (1.67)	8.82 (2.30)	0.80	0.420	0.18
Non-standard affect label	3.87 (1.47)	4.58 (2.33)	−1.59	0.117	−0.36
No affect label	0.95 (1.14)	0.61 (0.72)	1.57	0.121	0.36
Granularity					
Negative granularity	0.68 (0.12)	0.66 (0.14)	0.36	0.719	0.15
Positive granularity	0.65 (0.17)	0.65 (0.20)	−0.12	0.904	0.00

## Discussion

The current study examined the relationship between alexithymia and both interoceptive accuracy and sensibility in a non-clinical sample of young adults. We tested whether individuals who underwent a brief mindfulness-based body scan meditation demonstrated improved interoceptive accuracy and sensibility, as well as greater emotional awareness, relative to those in a control condition. While accounting for potential confounding variables, we found that the relationship between alexithymia and interoceptive accuracy in the current sample was best described as quadratic; elevated alexithymia was associated with having either relatively high or relatively low interoceptive accuracy. With regards to group differences in performance on interoceptive and emotional awareness tasks, we found that both the meditation and control groups showed significant increases in interoceptive accuracy from baseline to follow-up, with moderate effect sizes. Contrary to hypotheses, there were no group differences in degree of improvement. Also contrary to hypotheses, there were no group (meditation vs. control) differences in performance on affect labeling or emotional granularity.

We found a statistically significant quadratic relationship between interoceptive accuracy and total alexithymia, an effect which was moderate in magnitude. This effect was statistically significant for all alexithymia subscales, and strongest in effect for difficulty identifying feelings and difficulty describing feelings. Some existing research suggests that alexithymia may relate to reduced interoceptive accuracy (10). While this account is intuitive and supported by some empirical evidence, a negative relationship between alexithymia and interoceptive accuracy is not consistently reported (16, 19). The current findings highlight one potential source of inconsistency for these disparate findings in suggesting that the relationship between alexithymia and interoceptive accuracy may not be best categorized by a negative linear association, and rather, may be quadratic. In addition to individuals with elevated alexithymia that is associated with reduced interoceptive accuracy, a subset of individuals with heightened alexithymia may be characterized by increased interoceptive accuracy, which could also be maladaptive. Evidence from various clinical populations—briefly discussed in our introduction—supports this conjecture. This pattern may characterize other clinical populations as well. For example, hypervigilance toward pain is related to the development and maintenance of chronic pain conditions (41); however, chronic pain is also associated with elevated alexithymia (40). Likewise, negative outcomes may be associated with increased interoceptive accuracy in the case of panic disorders, where hypervigilance toward sensations of anxiety is thought to promote catastrophic interpretations, resulting in symptoms of panic (24).

To summarize, our findings indicate that greater interoceptive accuracy is not necessarily associated with better outcomes (e.g., emotional awareness); rather, a specific degree of interoceptive accuracy paired with neutral (vs. catastrophic) interpretations of interoceptive signals may be most adaptive. This is an empirical question for future research studies, which should continue investigating the relationship between alexithymia and interoceptive accuracy, as well as possible cognitive, clinical, and personality moderators of this relationship. This line of research has potential to shed light on developing and delivering targeted and personalized interventions to ameliorate the broad range of affective deficits associated with alexithymia. It is also important that future research replicate the findings presented in the current study, and using varied assessment approaches. As we discuss in more detail in the limitations section, there are limitations to the assessment approach adopted in this particular study. Replication and variety of methodological approach will help shed more light on the preliminary findings of a quadratic relationships between alexithymia and interoceptive accuracy reported in this study. In particular, recent guidelines have been published that will be helpful in guiding future research in this area (35).

The relationship between interoceptive sensibility and total alexithymia in the current study was best characterized as linear, although this was a considerably weaker effect, particularly for alexithymia subscales. A significant negative relationship between interoceptive sensibility and alexithymia has been reported in some (15, 16), but not all (18), empirical investigations of the linear relationship between these two variables. In the current study, interoceptive sensibility was quantified as participants' self-rated confidence in their accuracy detecting heart beats, which was impacted by self-report biases. Alexithymia is strongly related to psychological distress (42) and negative affect (43), which can result in a negative bias of one's abilities. Individuals with elevated alexithymia may be more likely to rate their abilities as poor, regardless of objective accuracy (42) and this may be particularly prominent when they are asked about confidence in areas concerning emotional and bodily awareness. We did not find a statistically significant relationship between externally oriented thinking and interoceptive sensibility. Although we cannot assess this in the current study, one possible explanation for these findings is that externally oriented thinking is less tightly linked to psychological distress compared to other alexithymia subscales (44), and thus less likely to be impacted by a negative self-bias. Moreover, other studies have failed to find a significant relationship between interoceptive sensibility and externally oriented thinking (16, 18), and the theoretical basis and psychometric properties of this construct have been questioned (45). Overall, there remains inconsistency in the relationship between alexithymia subscales and interoceptive sensibility. Future research is needed to more rigorously examine relationships between interoceptive sensibility and alexithymia (total and subscales), the potentially mediating role of negative affect, and using other indices of interoceptive sensibility—particularly those that minimize the confounds associated with self-report.

Contrary to our hypothesis, the mindfulness-based body scan intervention did not result in improvements in interoceptive accuracy above and beyond that of a control condition: both groups showed improvements in interoceptive accuracy, which were large in magnitude. These improvements are consistent with other studies that show a single, brief, mindfulness meditation can result in improved sensory awareness (36) and meaningful clinical change (37). These findings are inconsistent with several studies of small sample sizes (ns <41), which reported no statistically significant improvements in interoceptive accuracy among individuals in a meditation vs. control condition (46, 47). A recent study with a large sample size (*n* = 300) showed that adults enrolled in a 9-month mindfulness training program showed improvements in interoceptive accuracy, and that these improvements corresponded with reductions in alexithymia (6). Interestingly, the adults in that trial showed steady improvement in interoceptive accuracy over the duration of the 9-month training program, and the authors noted that long-term mindfulness training may be required for clinically meaningful and lasting improvements in interoceptive accuracy. Thus, the brief meditation intervention in the present study may have been insufficient in dose to cause measurable benefits in interoceptive accuracy, interoceptive sensibility or emotional awareness. With consideration to the quadratic relationship observed between interoceptive accuracy and alexithymia, additional research is needed to determine how to promote an adaptive degree of interoceptive accuracy using efficient intervention design, and with consideration to potential clinical moderators. Thus, ideally, treatment delivery might be best customized to individuals based on specific diagnoses or symptom profiles.

There were no overall changes in interoceptive sensibility from baseline to follow-up. However, we found a statistically significant interaction of time by group for interoceptive sensibility, which was moderate in effect. *Post-hoc* tests revealed that whereas those in the mindfulness intervention group showed small and statistically non-significant reduction in interoceptive sensibility from baseline to follow-up, those in the control group showed small and statistically non-significant improvement in interoceptive sensibility. These findings are inconsistent with some others, which show, for example, that mindfulness meditation training can result in improvements in interoceptive sensibility but not interoceptive accuracy (47), a pattern also found in expert meditators (48). These current findings should be interpreted with caution, as *post-hoc* comparisons were statistically non-significant, and these and other reported findings are certainly in need of replication; larger sample sizes are often required for adequate statistical power to accurately detect interactions, compared to main effects. Nonetheless, one possible explanation for the aforementioned significant interaction is that the body scan meditation led to increased awareness of the challenge of accurately perceiving interoceptive cues, resulting in decreased perceived accuracy on the heartbeat tracking task, relative to those in a control condition: Experts in the field of mindfulness meditation have argued that initially, such practices may increase awareness of one's deficits (49).

Contrary to our hypotheses, there were no significant group differences (meditation intervention vs. control) in affect labeling or emotional granularity. A brief mindfulness-based body scan meditation may be insufficient to increase emotional awareness, at least as assessed using the current paradigm. More interactive approaches might prove more useful for targetting specific alexithymia-related deficits in emotional awareness. For example, a recent study developed a novel body mapping intervention program, in which participants received education about affect and physiology, underwent guided emotion-focused body scan meditations, and completed digital mapping exersizes, visually depicting experienced emotions. When compared to a control condition of a body scan meditation alone, body mapping resulted in more precise and complex affect labeling, even in individuals with heightened alexithymia (34). Interactive and educational interventions may be superior to more passive, body scan meditations, such as that used in the current study, in improving affective labeling. Prior literature demonstrates the malleability of affect labeling and emotional granularity (31, 34), and additional research is needed to continue investigating effective methods of improving emotional awareness in the context of elevated alexithymia.

A number of methodological limitations are necessary to consider when interpreting the findings of this study. To account for the potential covariance of depression and anxiety on the relationship between alexithymia and interoceptive accuracy and sensibility, we excluded participants with a current or historical diagnosis of depression, anxiety or substance abuse. However, it is possible that sub-clinical symptoms impacted study findings. Future studies should control for these variables more rigorously. Further, as participants in the current study were healthy young adults, screened to ensure the absence of current or past psychopathology, variability in our constructs of interest (e.g., alexithymia, emotion granularity, and interoceptive awareness) was potentially limited. Participants completed a single practice trial of the heartbeat detection task prior to baseline assessment of interoceptive accuracy and sensibility; practice effects from baseline to follow-up may account in part for improved interoceptive accuracy in both the meditation and control groups. Future studies should administer a greater number of practice trials to better account for the potential confound of practice effects. The sample size for this study was informed by a power analysis of a single study examining the relationship between interoceptive accuracy and alexithymia, which reported a moderate negative association; this was likely to overestimate the expected effect size, and we were underpowered to detect statistically signficant smaller effects, particularly at the alexithymia subscale level.

It is important to note that heartbeat tracking tasks like Schandry's (14) have been criticized and their validity questioned (50). For example, participants can estimate their heartbeat in the absence of sensation. When participants are explicitly instructed to count only felt (and not estimated) heartbeats, interoceptive accuracy is reduced by 50% (35). While we took steps in the current study to remove non-interoceptive heartbeat detection cues (e.g., removing watches and clocks from room; instructing participants to rest with palms faced up on the table), participants were not explicitly instructed to report only felt heartbeats or to avoid using non-interoceptive signals (e.g., pulse) as information. Other covariates, including beliefs about heart rate and time estimation, were not assessed and could not be controlled for. Based on the conclusions of Desmedt et al. (35), interoceptive accuracy was likely overestimated in the current study. In the absence of such explicit instructions, heartbeat detection scores in the current study likely reflect a combination of actual detection, past knowledge of heartrate, or time estimation. A benefit of this paradigm is that it facilitates comparison with prior research done in this area, which has utilized a similar approach to assessing heartbeat detection. However, this has also likely contributed to the inconsistency of findings in this field. Considering this limitation, the current finding of a quadratic relationship between interoceptive accuracy and alexithymia must be considered preliminary. Future studies should examine this relationship using more rigorous methods, in line with recently published guidelines [cf. (35)], and assessing other domains of interoceptive accuracy.

Recent years have seen an explosion of research on alexithymia, a promising transdiagnostic construct with the potential shed light on socioemotional deficits across a variety of clinical phenomena and diagnostic groups. Our current work provides new insights on a particularly promising branch of this field, the relation between alexithymia and interoceptive accuracy and sensibility. These findings provide preliminary evidence that there may be more to the interplay of these two constructs than a simple negative, linear relationship. Specifically, in addition to the purported mechanism whereby interoceptive deficits contribute to elevated alexithymia, for some individuals, high levels of interoceptive accuracy might be associated with higher levels of alexithymia. These findings are preliminary and require replication in larger samples using recently published guidelines for assessing interoceptive accuracy (35). In addition, possible moderators, mediators, and direction of causality remain undetermined. We consider these important avenue for future research, particularly as findings carry implications for delivering targeted and personalized intervention to ameliorate affective deficits associated with alexithymia. Indeed, a “one size fits all” approach to ameliorating affective deficits associated with alexithymia may be inappropriate, given potential variability in interoceptive accuracy among those with elevated alexithymia. The current findings take steps toward providing a nuanced elucidation of the relationship between alexithymia and interoceptive accuracy and sensibility, while also setting forth a useful framework for future investigations.

## Data Availability Statement

The datasets generated for this study are available on request to the corresponding author.

## Ethics Statement

The studies involving human participants were reviewed and approved by Vanderbilt University IRB Review Board. The patients/participants provided their written informed consent to participate in this study. Written informed consent was obtained from the individual(s) for the publication of any potentially identifiable images or data included in this article.

## Author Contributions

RA and SP contributed conception and design of the study. RA, MS, and SB performed statistical analysis. RA wrote the first draft of the manuscript. MS and SB wrote sections of this manuscript. All authors contributed to the manuscript revision, read and approved the submitted version.

### Conflict of Interest

The authors declare that the research was conducted in the absence of any commercial or financial relationships that could be construed as a potential conflict of interest.
