# Supplementary material for: Quadratic Relationship Between Alexithymia and Interoceptive Accuracy, and Results From a Pilot Mindfulness Intervention
Source: Front Psychiatry. 2020 Mar 10;11:132. doi: 10.3389/fpsyt.2020.00132 (PMC7076086; doi:10.3389/fpsyt.2020.00132)
Supplement: Supplementary file 1 [file Data_Sheet_1.doc]

Supplemental Material A

*Hierarchical regression models of linear and quadratic relationships between alexithymia subscales and baseline interoceptive accuracy.*

|  | | *β* | *t* | *p* | R^2^ | *p* | *f^2^* | Change | |
| --- | --- | --- | --- | --- | --- | --- | --- | --- | --- |
|  |  |  |  |  |  |  |  | ΔR^2^ | *p(*ΔF*)* |
| TAS-20 DDF | |  |  |  |  |  |  |  |  |
| Step 1 | |  |  |  | 0.03 | 0.373 | 0.03 | 0.03 | 0.373 |
|  | BMI | -0.18 | -1.42 | 0.162 |  |  |  |  |  |
|  | Gender | -0.06 | -0.50 | 0.626 |  |  |  |  |  |
| Step 2 | |  |  |  | 0.04 | 0.482 | 0.04 | 0.01 | 0.483 |
|  | BMI | -0.16 | -1.28 | 0.205 |  |  |  |  |  |
|  | Gender | -0.06 | -0.47 | 0.640 |  |  |  |  |  |
|  | IA_b_ | -0.09 | -0.71 | 0.483 |  |  |  |  |  |
| Step 3 | |  |  |  | **0.14** | **0.034** | **0.16** | **0.11** | **.005** |
|  | BMI | -0.11 | -0.89 | 0.378 |  |  |  |  |  |
|  | Gender | 0.04 | 0.31 | 0.759 |  |  |  |  |  |
|  | **IA_b_** | **-2.39** | **-2.97** | **0.004** |  |  |  |  |  |
|  | **IA_b_^2^** | **2.32** | **2.89** | **0.005** |  |  |  |  |  |
| TAS-20 DIF | |  |  |  |  |  |  |  |  |
| Step 1 | |  |  |  | 0.07 | 0.084 | 0.08 | 0.07 | 0.084 |
|  | **BMI** | **-0.28** | **-2.25** | **0.028** |  |  |  |  |  |
|  | Gender | -0.06 | -0.47 | 0.641 |  |  |  |  |  |
| Step 2 | |  |  |  | 0.08 | 0.140 | 0.09 | 0.01 | 0.450 |
|  | **BMI** | **-0.26** | **-2.10** | **0.040** |  |  |  |  |  |
|  | Gender | -0.06 | -0.45 | 0.656 |  |  |  |  |  |
|  | IA_b_ | -0.09 | -0.76 | 0.450 |  |  |  |  |  |
| Step 3 | |  |  |  | **0.13** | **0.046** | **0.15** | **0.06** | **0.042** |
|  | BMI | -0.22 | -1.80 | 0.076 |  |  |  |  |  |
|  | Gender | -0.02 | 0.12 | 0.904 |  |  |  |  |  |
|  | **IA_b_** | **-1.75** | **-2.16** | **0.034** |  |  |  |  |  |
|  | **IA_b_^2^** | **1.67** | **2.07** | **0.042** |  |  |  |  |  |
| TAS-20 EOT | |  |  |  |  |  |  |  |  |
| Step 1 | |  |  |  | 0.01 | 0.609 | 0.01 | .01 | 0.609 |
|  | BMI | 0.10 | 0.79 | 0.433 |  |  |  |  |  |
|  | Gender | -0.04 | -0.33 | 0.740 |  |  |  |  |  |
| Step 2 | |  |  |  | 0.02 | 0.792 | 0.02 | 0.00 | 0.815 |
|  | BMI | 0.10 | 0.74 | 0.464 |  |  |  |  |  |
|  | Gender | -0.04 | -0.34 | 0.737 |  |  |  |  |  |
|  | IA_b_ | 0.03 | 0.24 | 0.815 |  |  |  |  |  |
| Step 3 | |  |  |  | **0.05** | **0.112** | **0.05** | **0.09** | **0.012** |
|  | BMI | 0.15 | 1.16 | 0.252 |  |  |  |  |  |
|  | Gender | 0.05 | 0.37 | 0.717 |  |  |  |  |  |
|  | **IA_b_** | **-2.08** | **-2.52** | **0.014** |  |  |  |  |  |
|  | **IA_b_^2^** | **2.12** | **2.58** | **0.012** |  |  |  |  |  |

*Note:* BMI = Body Mass Index; IA_b_ = Baseline Interoceptive Accuracy. Statistically significant values (*p* < .05) bolded.

Supplemental Material B.

*Hierarchical regression models of linear and quadratic relationships between alexithymia subscales* and baseline interoceptive sensibility.

|  | | *β* | *t* | *p* | R^2^ | *p* | *f^2^* | Change | | | |
| --- | --- | --- | --- | --- | --- | --- | --- | --- | --- | --- | --- |
|  |  |  |  |  |  |  |  | ΔR^2^ | | *p(*ΔF*)* | |
| TAS-20 DDF | |  |  |  |  |  |  | |  | |  |
| Step 1 | |  |  |  | -0.00 | 0.395 | 0.00 | | 0.03 | | 0.395 |
|  | BMI | -0.18 | -1.37 | 0.175 |  |  |  | |  | |  |
|  | Gender | -0.06 | -0.48 | 0.635 |  |  |  | |  | |  |
| Step 2 | |  |  |  | 0.03 | 0.178 | 0.03 | | 0.05 | | 0.082 |
|  | BMI | -0.19 | -1.53 | 0.131 |  |  |  | |  | |  |
|  | Gender | -0.02 | -0.13 | 0.895 |  |  |  | |  | |  |
|  | IA_b_ | -0.22 | -1.77 | 0.082 |  |  |  | |  | |  |
| Step 3 | |  |  |  | 0.01 | 0.299 | 0.01 | | 0.00 | | 0.929 |
|  | BMI | -0.20 | -1.51 | 0.136 |  |  |  | |  | |  |
|  | Gender | -0.02 | -0.13 | 0.897 |  |  |  | |  | |  |
|  | IA_b_ | -0.27 | -0.43 | 0.668 |  |  |  | |  | |  |
|  | IA_b_^2^ | -0.06 | 0.09 | 0.929 |  |  |  | |  | |  |
| TAS-20 DIF | |  |  |  |  |  |  | |  | |  |
| Step 1 | |  |  |  | 0.07 | 0.089 | 0.08 | | 0.07 | | 0.089 |
|  | BMI | -0.28 | -2.23 | 0.029 |  |  |  | |  | |  |
|  | Gender | -0.06 | -0.48 | 0.634 |  |  |  | |  | |  |
| **Step 2** | |  |  |  | **0.12** | **0.037** | **0.14** | | 0.05 | | 0.057 |
|  | BMI | -0.30 | -2.42 | 0.018 |  |  |  | |  | |  |
|  | Gender | -0.01 | -0.10 | 0.919 |  |  |  | |  | |  |
|  | IA_b_ | -0.23 | -1.94 | 0.057 |  |  |  | |  | |  |
| Step 3 | |  |  |  | 0.12 | 0.076 | 0.14 | | 0.00 | | 0.826 |
|  | BMI | -0.30 | -2.40 | 0.019 |  |  |  | |  | |  |
|  | Gender | -0.01 | -0.10 | 0.921 |  |  |  | |  | |  |
|  | IA_b_ | -0.37 | -0.59 | 0.555 |  |  |  | |  | |  |
|  | IA_b_^2^ | -0.14 | 0.22 | 0.826 |  |  |  | |  | |  |
| TAS-20 EOT | |  |  |  |  |  |  | |  | |  |
| Step 1 | |  |  |  | 0.01 | 0.632 |  | | 0.01 | | 0.632 |
|  | BMI | 0.10 | 0.80 | 0.429 |  |  |  | |  | |  |
|  | Gender | -0.04 | -0.29 | 0.776 |  |  |  | |  | |  |
| Step 2 | |  |  |  | 0.03 | 0.528 |  | | 0.02 | | 0.262 |
|  | BMI | 0.09 | -0.71 | 0.483 |  |  |  | |  | |  |
|  | Gender | -0.01 | -0.06 | 0.952 |  |  |  | |  | |  |
|  | IA_b_ | -0.14 | -1.13 | 0.262 |  |  |  | |  | |  |
| Step 3 | |  |  |  | 0.04 | 0.672 |  | | 0.00 | | 0.702 |
|  | BMI | 0.08 | 0.63 | 0.534 |  |  |  | |  | |  |
|  | Gender | -0.01 | -0.06 | 0.954 |  |  |  | |  | |  |
|  | IA_b_ | -0.39 | -0.60 | 0.553 |  |  |  | |  | |  |
|  | IA_b_^2^ | 0.25 | 0.38 | 0.702 |  |  |  | |  | |  |

*Note:* BMI = Body Mass Index; IS_b_ = Baseline Interoceptive Sensibility. Significant values bolded.
